# Cell-to-cell communication mediates glioblastoma progression in *Drosophila*

**DOI:** 10.1242/bio.053405

**Published:** 2020-09-29

**Authors:** Marta Portela, Teresa Mitchell, Sergio Casas-Tintó

**Affiliations:** 1 Molecular, Cellular and Developmental Neurobiology Department, Instituto Cajal-CSIC, Av. del Doctor Arce, 37, 28002 Madrid, Spain; 2 Department of Biochemistry and Genetics, La Trobe Institute for Molecular Sciences, La Trobe University, 3086 Melbourne, Australia

**Keywords:** Glia, Cancer, Glioblastoma, Tumour microtubes, JNK, Neurodegeneration

## Abstract

Glioblastoma (GB) is the most aggressive and lethal tumour of the central nervous system (CNS). GB cells grow rapidly and display a network of projections, ultra-long tumour microtubes (TMs), that mediate cell to cell communication. GB-TMs infiltrate throughout the brain, enwrap neurons and facilitate the depletion of the signalling molecule wingless (Wg)/WNT from the neighbouring healthy neurons. GB cells establish a positive feedback loop including Wg signalling upregulation that activates cJun N-terminal kinase (JNK) pathway and matrix metalloproteases (MMPs) production, which in turn promote further TMs infiltration, GB progression and neurodegeneration. Thus, cellular and molecular signals other than primary mutations emerge as central players of GB. Using a *Drosophila* model of GB, we describe the temporal organisation of the main cellular events that occur in GB, including cell-to-cell interactions, neurodegeneration and TM expansion. We define the progressive activation of JNK pathway signalling in GB mediated by the receptor Grindelwald (Grnd) and activated by the ligand Eiger (Egr)/TNFα produced by surrounding healthy brain tissue. We propose that cellular interactions of GB with the healthy brain tissue precede TM expansion and conclude that non-autonomous signals facilitate GB progression. These results contribute to deciphering the complexity and versatility of these incurable tumours.

## Abstract (precis)

**Summary:** Using a *Drosophila* model of glioblastoma (GB), we describe the temporal organisation of the main cellular events that occur in GB, including cell-to-cell interactions, neurodegeneration and tumour microtubes expansion.

## INTRODUCTION

Glioblastoma multiforme (GB) is the most frequent and aggressive primary malignant brain tumour, with an incidence of three per 100,000/year (Gallego, 2015). GB patients’ median survival is 12–15 months, with less than 5% chance of survival after 5 years (Gallego, 2015; Louis et al., 2016; McGuire, 2016; Rogers et al., 2018). The causes of GB are under debate (McGuire, 2016), 5% of the patients develop GB after a low grade astrocytoma (Alifieris and Trafalis, 2015) and the most frequent mutations include gain of function of the epidermal growth factor receptor (EGFR) (97%) and the phosphatidylinositol-3 kinase (PI3K)/phosphatase and tensin homologue (PTEN) pathways (88%) (Hayden, 2010). The diagnosis, and therefore the treatment of GB, requires a mutation analysis taking into account the high frequency of clones within the same primary GB (Wick et al., 2018). Temozolomide (TMZ) is the only treatment for GB, however, recent discoveries restrict the use of TMZ in GB patients depending on the methylation status of methylguanine DNA methyltransferase (MGMT) (Wick et al., 2018). Moreover, among other mutations, *Isocitrate dehydrogenase* (*IDH*) defines the nature and features of GB (Waitkus et al., 2018) together with molecular alterations including 1p/10q deletions and *tumour suppressor protein 53* (*TP53*) and *alpha thalassemia/mental retardation* (*ATRX*) mutations (Miller et al., 2017; Waitkus et al., 2018). The genetic and molecular heterogeneity complicate the diagnosis and treatment of these fatal brain tumours.

EGFR mutant forms show constitutive kinase activity that chronically stimulates Ras signalling to drive cell proliferation and migration (Furnari et al., 2007; Maher et al., 2001). Other common genetic lesions include loss of the lipid phosphatase PTEN, which antagonises the PI3K signalling pathway, and mutations that activate PI3KCA, which encodes the p110a catalytic subunit of PI3K (Furnari et al., 2007; Maher et al., 2001). GBs often show constitutively active Akt, a major PI3K effector. Multiple mutations that coactivate EGFR-Ras and PI3K/Akt pathways are required to induce a glioma (Holland, 2000). In *Drosophila*, a combination of EGFR and PI3K mutations effectively causes a glioma-like condition that reproduces the features of human gliomas including glia expansion, brain invasion, neuron dysfunction, synapse loss and neurodegeneration. This model involves the co-overexpression of constitutively active forms of EGFR (dEGFR^λ^) and an activated membrane-localised version of the PI3K catalytic subunit p110α/PI3K92E (dPI3K92E^CAAX^) under Gal4 UAS control, specifically driven in the glial cells by means of *repo-Gal4* (Brand and Perrimon, 1993; Casas-Tintó et al., 2017; Read et al., 2009).

The recent discovery of a network of ultra-long tumour microtubes (TMs) in GB (Osswald et al., 2015), also known as cytonemes in *Drosophila* (Casas-Tintó and Portela, 2019)*,* improves our understanding of GB progression and therapy resistance (Osswald et al., 2016). In GB, this network of TMs mediates cell-to-cell communication. TMs are actin-based filopodia that infiltrate into the brain and reach long distances within the brain (Osswald et al., 2015). TMs are required in GB cells to mediate Wingless (Wg)/WNT signalling imbalance among neurons and GB cells. Wg/WNT signalling is increased in GB cells to promote tumoural progression, at the expense of neuronal Wg signalling, which results in neurodegeneration and lethality (Arnés and Casas Tintó, 2017; Casas-Tintó and Portela, 2019; Portela et al., 2019). Another recent study has shown that GB produces ImpL2, an antagonist of the insulin pathway that targets neighbouring neurons and causes mitochondrial disruption as well as synapse loss. *Rheb* overexpression in neurons, which results in activation of the PI3K pathway, prevents mitochondrial disruption as well as synapse loss and the reduction in life span caused by GB (Jarabo et al., 2020).

The central role of TMs in GB biology has emerged as a fundamental mechanism for GB rapid and lethal progression; thus, it is an attractive field of study towards potential GB treatments. However, the molecular mechanisms underlying the expansion of TMs and the signalling pathways mediating TM infiltration are still poorly understood.

The Jun-N-terminal Kinase (JNK) pathway is a hallmark of GB cells that is associated to glial proliferation and stem-like status, and currently it is a pharmacological target for GB (Matsuda et al., 2012). Moreover, the JNK pathway is the main regulator of matrix metalloproteases (MMPs) expression and cell motility in many organisms and tissues including tumours like GB (Cheng et al., 2012; Ispanovic and Haas, 2006; Lee et al., 2009; Portela et al., 2019; Uhlirova and Bohmann, 2006; Zeigler et al., 1999).

MMPs are a family of endopeptidases capable of degrading the extracellular matrix (ECM). Members of the MMP family include the ‘classical’ MMPs, the membrane-bound MMPs (MT-MMPs), the ADAMs (a disintegrin and metalloproteinase; adamlysins) and the ADAMTS (a disintegrin and metalloproteinase with thrombospondin motif). There are more than 20 members in the MMP and ADAMTS family including the collagenases, gelatinases, stromelysins, some elastases and aggrecanases (Malemud, 2006). The vertebrate MMPs have genetic redundancy and compensation, they have overlapping substrates, and pharmacological inhibitors are non-specific. There are two orthologues to human MMPs in *Drosophila*, *MMP1* and *MMP2*. MMP1 is secreted and MMP2 is membrane-anchored (Page-McCaw et al., 2003). However, recent reports propose that products of both genes are found at the cell surface and released into media, and that GPI-anchored MMPs promote cell adhesion when they become inactive. Moreover, the two MMPs cleave different substrates, suggesting that this is the important distinction within this small MMP family (LaFever et al., 2017). MMPs are upregulated in several tumours, including GBs. Cancer cells produce MMPs to facilitate tumour progression and invasiveness and MMPs upregulation in GB is associated with the diffuse infiltrative growth and have been proposed to play a role in GB cell migration and infiltration (de Lucas et al., 2016; Veeravalli and Rao, 2012; reviewed in Nakada et al., 2003). MMPs are upregulated in human GB cell-lines and biopsies as compared with low-grade astrocytoma (LGA) and normal brain samples (Hagemann et al., 2012, 2010). In particular, among the 23 MMPs present in humans, MMP9, MMP2 and MMP14 are directly implicated in growth and invasion of GB cells (Munaut et al., 2003).

WNT induces MMPs expression during development and cancer (Lowy et al., 2006; Lyu and Joo, 2005; Page-McCaw et al., 2003; Roomi et al., 2017; Uraguchi et al., 2004) associated to cell migration and metastasis. Specifically, in human GB, MMP2 expression and their infiltrative properties correlate with Wnt5 (Kamino et al., 2011; Roth et al., 2000) and MMP9 is upregulated upon EGFR activity (Chen et al., 2016).

In consequence, MMPs upregulation in GB is an indicator of poor prognosis (Yamamoto et al., 2002) and the study of the mechanisms mediated by MMPs is relevant for the biology of GB, and cancer in general. GB cells project TMs that cross the ECM and infiltrate in the brain to reach territories distant from the primary GB site (Osswald et al., 2015, 2016). We have previously demonstrated that GB cells activate the JNK pathway and accumulate MMPs (MMP1 and 2). MMPs contribute to TMs expansion in the brain and facilitate Wg/WNT signalling in GB cells through the receptor Frizzled1 (Fz1). Moreover, Wg/WNT signalling mediates JNK activation in GB cells to continue with MMPs production and TMs infiltration process. We hypothesise that the founder mutations in GB (PI3K and EGFR) initiate the process with the expansion of the TMs; afterwards, the system self-perpetuates (TMs-Fz1/Wg-JNK-MMPs-TMs) to facilitate GB progression and infiltration in the brain (Portela et al., 2019).

We have recently shown that JNK pathway activation mediated by the receptor Grnd is a requirement for GB progression, TMs expansion and Fz1-mediated Wg depletion from neurons. In turn, Wg pathway upregulation in GB induces JNK activity in GB cells that mediate the production of MMPs. Moreover, *MMPs* knockdown in GB cells is sufficient to rescue neurodegeneration and premature death caused by GB (Portela et al., 2019). However, the molecular mechanisms by which JNK pathway is activated in GB cells remain unknown.

Here, we investigated the mechanism by which the JNK signalling pathway is activated in GB cells. Egr activates the JNK pathway in GB cells through the specific receptor Grnd, highlighting again the contribution of communication signals between healthy brain tissue and GB cells to the progression of the disease (Jarabo et al., 2020; Portela et al., 2019). Through transcriptional and immunofluorescence analysis of Egr in GB, we found that Egr localises mostly into the brain tissue that includes neurons and neuroblasts, and a smaller fraction of Egr is present in healthy glial cells. However, in GB brains there is a shift of Egr from the surrounding healthy tissue towards GB cells. These results suggest that Egr is expressed by non-tumoural brain tissue, but it is accumulated in tumoural cells and activates the JNK pathway. In consequence, GB cells produce MMPs that facilitate TM infiltration and GB progression. At the cellular level, we analyse three aspects required for GB progression: TMs expansion, GB cell number increase and synapse loss in the surrounding neurons and propose a timeline for these events in GB progression.

## RESULTS

### Combined activation of EGFR and PI3K pathways is required for GB progression

The *Drosophila* GB model reproduces the main features of the disease including the expansion of TMs. Cytonemes and TMs share multiple features (Casas-Tintó and Portela, 2019; Portela et al., 2019) and can be visualised with green/red fluorescent membrane tags (e.g. CD8-GFP or myr-RFP), or cytoneme components including signalling proteins such as Ihog (Ihog-RFP). Membrane markers are expressed under the control of the glial specific enhancer *repo-Gal4* (Casas-Tintó et al., 2017). Therefore, only glial cells are marked with RFP (red), and in the *Drosophila* brain the unlabelled surrounding cells are mostly neuroblasts and neurons that are marked with the neuron specific antibody anti-horseradish peroxidase (Hrp) in green (Fig. S1A) (Portela et al., 2019).

WNT signalling is a hallmark in gliomagenesis associated with the proliferation of stem-like cells in human GBs (Kahlert et al., 2015). We have previously shown that GB cells expand a network of TMs that accumulate the Wg receptor Fz1, and deplete Wg from the surrounding neurons, a mechanism that leads to activation of Wg signalling in these tumoural cells (Portela et al., 2019).

Concomitant activation of both PI3K and EGFR signalling pathways is necessary to induce a glioma (Read et al., 2009). First, we sought to clarify the separate contribution of PI3K and EGFR signalling pathways to GB progression, and the associated phenotypes including Wg/Fz1 abnormal distribution and signalling activation in GB cells. Fz1 localisation in the TMs is key to trigger Wg signalling and GB cell number increase. We first induced constitutive activation of EGFR or PI3K/PTEN pathways independently in glial cells, and using conventional immunofluorescence staining, we assessed the presence of Fz1 receptor in glial membranes with a specific monoclonal antibody previously validated (Portela et al., 2019). Fz1 receptor signal is localised homogeneously across the brain in control samples (Fig. S1B). However, in the GB model (PI3K+EGFR), Fz1 accumulates in TMs (Fig. S1C) (Portela et al., 2019). To determine if expression of constitutively active forms of PI3K or EGFR independently is sufficient to trigger TMs network expansion and Fz1 localisation in the TMs, we expressed *Drosophila PI3K* or *EGFR* (*UAS-dp110^CAAX^* or *UAS-TOR-DER^CA^*) in glial cells driven by *repo-Gal4* and stained with Fz1 antibody. The results show that Fz1 receptor signal is localised homogeneously across the brain (Fig. S1D,E) similar to control samples, and the glial TM network does not expand as in GB (compare Fig. S1D,E to Fig. S1B,C), although there are some morphological differences in the glial membranes upon *EGFR* overexpression compared to the control (Fig. S1B,E). These data suggest that the activation of both pathways together is necessary for the expansion of the TMs network and Fz1 localisation in the TMs.

PI3K and EGFR pathways converge in *dMyc* expression and *dMyc* expression is required for GB development (Annibali et al., 2014; Read et al., 2009; Tateishi et al., 2016; Wang et al., 2017). Thus, to determine if *dMyc* is sufficient to trigger TMs network and activate the Wg pathway, we ectopically overexpressed *dMyc* (*UAS-dMyc*) in glial cells that are labelled with ihog-RFP (*repo-Gal4*), and stained the brains with anti-Fz1 to detect the Wg receptor (Fig. S1F), or anti-Wg to detect the ligand Wg (Fig. S1G–I). Wg targets are indicators of Wg/Fz1 activity in the recipient cell. Armadillo/β-Catenin is a cytoplasmic protein which, upon activation of Wg pathway, translocates into the nucleus and activates transcription of target genes (Arnés and Casas Tintó, 2017; Klaus and Birchmeier, 2008). To determine whether ectopic overexpression of *dMyc* in glial cells is sufficient to trigger Wg/Fz1 signalling in GBs, we used an anti-Armadillo (Arm) antibody, which identifies its cytoplasmic inactive form (Cyt-Arm; Portela et al., 2019) (Fig. S1J–L). The confocal images show no morphological evidence of GB formation (Fig. S1F compare to GB in Fig. S1C). Moreover, Fz1, Wg or Cyt-Arm protein distribution resemble control brains (Fig. S1B,F,G,I,J,L). Taking these results together, we conclude that *dMyc* overexpression is not sufficient to reproduce the features of the GB. These results suggest that upregulation of both PI3K and EGFR signalling pathways in glial cells is necessary for the expansion of glial TMs, Fz1 localisation in TMs and activation of the Wg pathway to reproduce GB features.

**Fig. 1.**
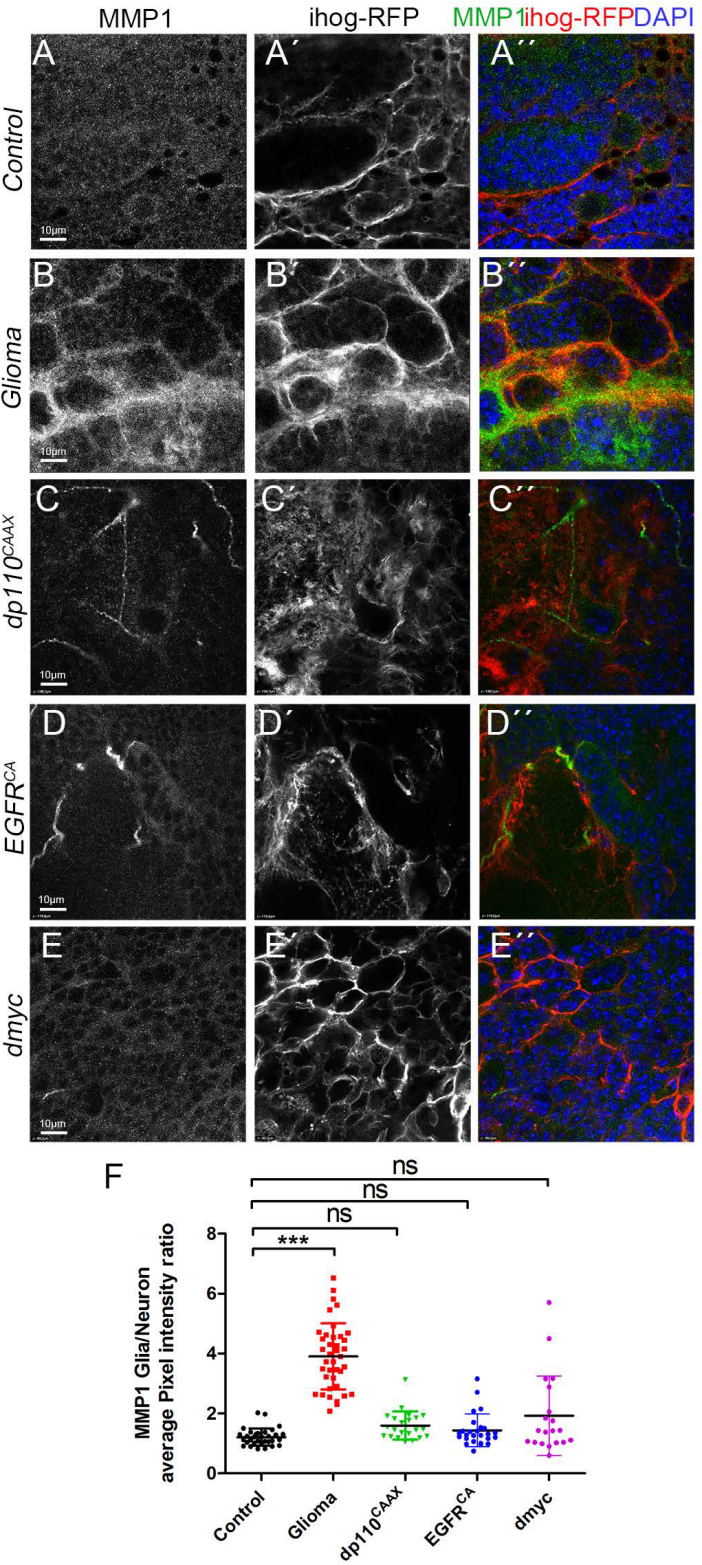
**Independent constitutive activation of PI3K or EGFR or ectopic *dmyc* are not responsible for MMP1 accumulation in GB.** Brain sections from third-instar larvae displayed at the same scale. Glia are labelled with *UAS-ihog-RFP* (gray or red in the merge) driven by *repo-Gal4* to visualise active cytonemes/ TM structures in glial cells and stained with MMP1 (gray or green in the merge). (A) MMP1 (gray or green in the merge) is homogeneously distributed in control sections, with a slight accumulation in the cytonemes. (B) In Glioma brains, MMP1 accumulates in the TMs and specifically in the TM projections that are in contact with the neuronal clusters. (C–E) MMP1 (gray or green in the merge) is homogeneously distributed in (C) *dp110^CAAX^*, (D)*TOR-DER^CA^* and (E) *dmyc* sections with a slight accumulation in the cytonemes similar to controls. Nuclei are marked with DAPI (blue). (F) Quantification of MMP1 staining ratio between ihog^+^ and ihog^–^ domains. Data from three independent experiments, *n*>10 samples analysed for each genotype per experiment. Kruskal–Wallis test with Dunn’s post-hoc test. Error bars show mean±s.d.; ***P<0.0001 or ns for non-significant. Scale bar size is indicated in all figures. The expression system was active, and the GB induced, during the whole development including both embryonic and larval stages in all experiments in this figure. Genotypes: (A) *UAS-lacZ/repo-Gal4, UAS-ihog-RFP;* (B) *UAS-dEGFR^λ^, UAS-dp110^CAAX^;; repo-Gal4, UAS-ihog-RFP;* (C) *UAS-dp110^CAAX^;; repo-Gal4, UAS-ihog-RFP;* (D) *UAS-TOR-DER^CA^;; repo-Gal4, UAS-ihog-RFP;* (E) *repo-Gal4, UAS-ihog-RFP/UAS-dmyc*.

We have previously shown that MMPs are upregulated in the *Drosophila* model of GB and that the TM infiltration through the brain is mediated by their activity (Portela et al., 2019). Next, we explored if the independent activation of EGFR or PI3K is sufficient to induce the upregulation of MMPs and the epistatic relations behind *MMPs* upregulation. To determine MMP production and localisation, we used a specific monoclonal antibody against *Drosophila* MMP1. The confocal images show that MMP1 is homogeneously distributed across the brain in control samples with a slight accumulation in the Ihog+ projections (Fig. 1A), and the quantifications revealed an increase in the MMP1 glia/neuron ratio in the TMs of GB samples (Fig. 1B). Consistent with the results shown above, MMP1 shows a homogeneous distribution through the brain, also with a slight accumulation in the Ihog+ projections, upon constitutive activation of PI3K (*dp110^CAAX^*) or EGFR (*TOR-DER^CA^*) in glial cells (Fig. 1C,D, quantified in F), comparable to the controls (Fig. 1A). Besides, *dMyc* overexpression in glial cells did not cause significant changes in MMPs localisation (Fig. 1E,F). Taking these results together, independent overexpression of *EGFR*, *PI3K* or *dMyc* is not sufficient to reproduce the features of GB. These results suggest that the combined activity of PI3K and EGFR pathways are necessary to activate a downstream pathway responsible for the expansion of TMs and MMP1 accumulation in GB cells; and *dMyc* overexpression is not sufficient to cause these phenotypes.

### Non-autonomous activation of JNK pathway in GB

In addition to Wg pathway, GB cells activate JNK pathway to maintain the stem-like cells, which has become a pharmacological target for the treatment of GB (Matsuda et al., 2012). Moreover, we have previously shown that JNK signalling activation, mediated by the receptor Grnd, is necessary in GB cells for TMs expansion and tumour progression (Portela et al., 2019). However, the specific mechanism of Grnd receptor activation is still unknown. A recent study using gene-expression profiling identified genes that were correlated significantly with the overall survival of patients with GB (Hsu et al., 2019). 104 genes were identified, which are common between patients with GB and those with low grade gliomas and can be used as core genes related to patient survival. Of these, ten genes (CTSZ, EFEMP2, ITGA5, KDELR2, MDK, MICALL2, MAP 2 K3, PLAUR, SERPINE1 and SOCS3) can potentially classify patients with gliomas into different risk groups. Among these pathways, the TNF-α signalling pathway stands out. Four genes from this ten-gene group (MAP 2 K3, PLAUR, SERPINE1 and SOCS3) are involved in TNFα signalling, and they might have potential prognostic value for patients with GB (Hsu et al., 2019).

**Fig. 2.**
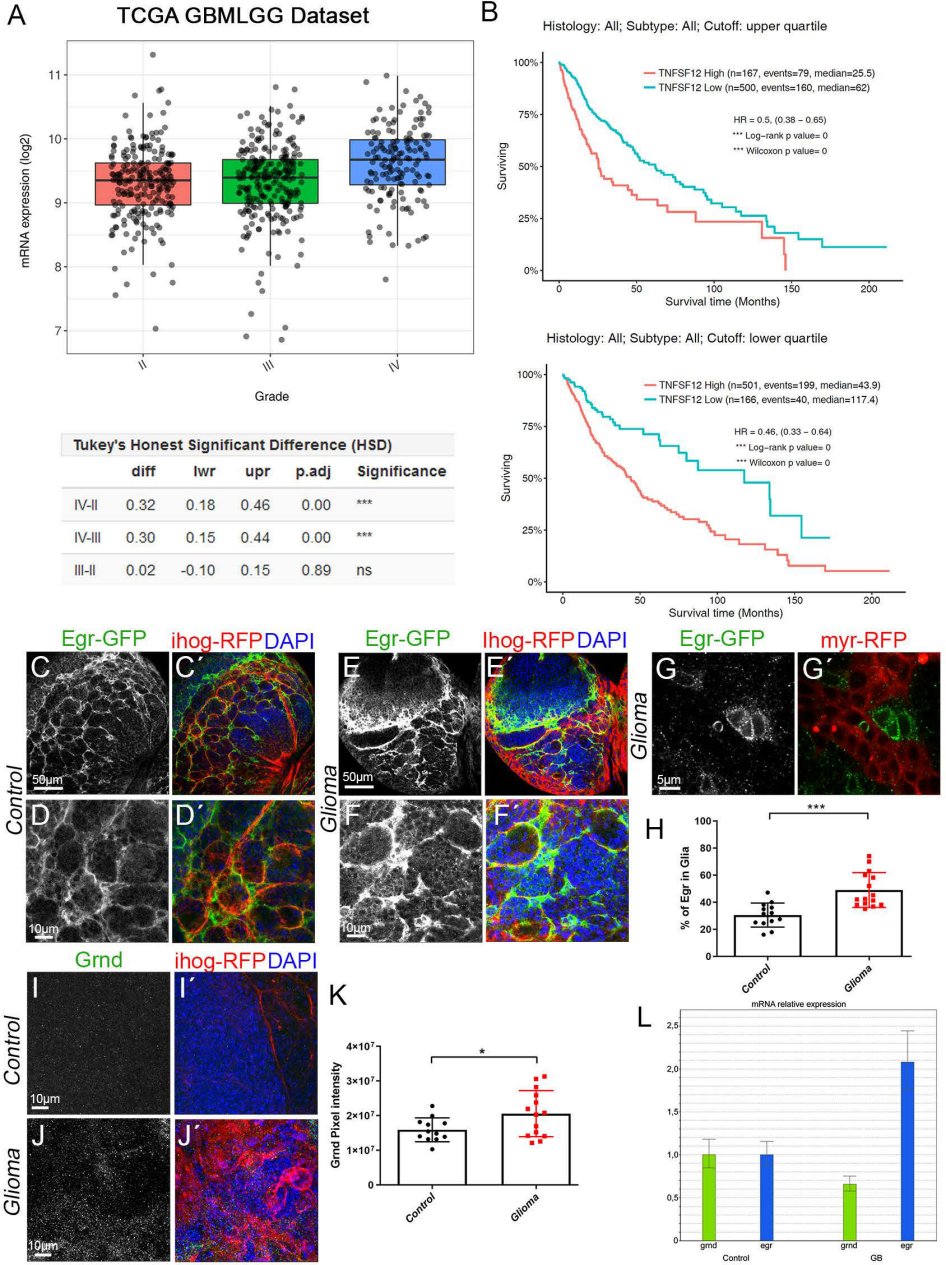
**Egr re-localises from neuron to glia and its receptor Grindelwald is accumulated in GB.** (A,B) *In silico* analysis of the data from the TCGA dataset from GlioVis (http://gliovis.bioinfo.cnio.es/) showing that TNFSF12 is highly upregulated in several tumours of the CNS of glial origin, including GB (grade IV), the statistical analysis extracted from the database is shown in the table below the graph (A). *In silico* analysis of TCGA data indicating the survival of patients with high or low TNFSF12 expression. Upper graph shows the upper quartile and the graph below shows the lower quartile (B). (C-J′) Brains from third-instar larvae. Glia are labelled with *UAS-Ihog-RFP* (gray or red in the merge) driven by *repo-Gal4* to visualise active cytonemes/TM structures in glial cells. Egr protein is visualised by GFP staining (green) of a transgenic *Drosophila* line in which the endogenous *egr* gene is GFP tagged (Egr-GFP protein fusion reporter). (C,D) In control brain sections, 30% of Egr-GFP (gray or green in the merge) signal localised in glial cells while most Egr-GFP signal (70%) localised in the surrounding cells that are in close contact with glial cells. (E–G) In glioma brain sections, Egr-GFP (green) signal shifts and 50% of the GFP signal localised in glioma cells and the remaining 50% localised in the healthy surrounding cells. Higher magnification image of a glioma brain section is shown in (G). (H) Quantification of the percentage of Egr-GFP localised in glial cells in control and glioma samples. (I,J) Grnd staining (gray or green in the merge) in control and glioma samples. (K) Quantification of the Grnd pixel intensity in control and glioma samples. Nuclei are marked with DAPI (blue). (L) qPCRs with RNA extracted from control and glioma larval brains showing a twofold increase of the transcription (mRNA levels) of *egr*. Data from two independent experiments, *n*>10 samples analysed for each genotype per experiment. Two-tailed *t*-test with Welch’s correction. Error bars show mean±s.d.; * *P*<0.05, ***P≤0.0001. Scale bar size is indicated in all figures. The expression system was active, and the GB induced, during the whole development including both embryonic and larval stages in all experiments in this figure. Genotypes: (C,D) Egr-GFP; *repo-Gal4, ihog-RFP/UAS-lacZ*; (E,F) *UAS-dEGFR^λ^, UAS-dp110^CAAX^;Egr-GFP; repo-Gal4, UAS-ihog-RFP;* (G) *UAS-dEGFR^λ^, UAS-dp110^CAAX^; Gal80^ts^; Egr-GFP; repo-Gal4, myr-RFP;* (I) *repo-Gal4, ihog-RFP/UAS-lacZ*; (J) *UAS-dEGFR^λ^, UAS-dp110^CAAX^;; repo-Gal4, UAS-ihog-RFP*.

TNF-like weak inducer of apoptosis (TWEAK, also known as TNFSF12) is a ligand of the TNF family (Chicheportiche et al., 1997). TNFSF12 preferentially activates non-canonical NF-κB and promotes the invasive properties of glioma cells (Cherry et al., 2015). To determine the contribution of TNFSF12 to GB, we analysed the data from The Cancer Genome Atlas (TCGA) dataset from GlioVis (http://gliovis.bioinfo.cnio.es/). *In silico* analysis showed that TNFSF12 gene is highly upregulated in several samples of CNS tumours, including GB (grade IV) (Fig. 2A). Accordingly, *TNFSF12* expression has a negative implication to the overall survival of patients with gliomas (Fig. 2B).

Due to the relevance of JNK pathway and TNFα ligand in GB prognosis and progression, and to provide mechanistic insight into how the JNK pathway and MMPs are activated in GB and whether this is an autonomous or non-autonomous regulatory mechanism, we decided to study Egr, the solely *Drosophila* orthologue of the ligand for the mammalian TNF receptor signalling pathway (Igaki et al., 2002; Moreno et al., 2002) and its receptor Grnd (Andersen et al., 2015), in the *Drosophila* GB model.

### Egr re-localises from neurons to glia in GB

Next, we investigated whether Egr, a ligand of JNK pathway, is involved in in GB progression. We first assessed Egr protein localisation. To monitor Egr protein distribution, we used a previously validated transgenic *Drosophila* line (Doupé et al., 2018) which carries a transposon insertion that leads to a protein fusion of endogenous Egr tagged with GFP (Nagarkar-Jaiswal et al., 2015; Doupé et al., 2018). The results from confocal microscopy show that 70% of Egr-GFP signal is localised in the tissue surrounding the glial cells and 30% of Egr-GFP signal is localised in the glia (Fig. 2C,D and H). However, in GB samples there is a shift of Egr-GFP signal from the surrounding tissue towards glial cells (∼50%) (Fig. 2E–H). We validated the Egr-GFP fusion protein with anti-MMP2 staining as a target of the JNK pathway in glial cells (Portela et al., 2019). The confocal images show that GFP signal coincides with MMP2 signal, suggesting that Egr-GFP is a functional tool (Fig. S2A). Next, we monitored the expression pattern of the JNK receptor Grnd in GB and control samples, with a specific antibody previously validated (Andersen et al., 2015). The quantification of Grnd signal shows an increase of Grnd protein in the membrane of GB cells (Fig. 2I–K). The abnormal distribution of Egr and Grnd in GB brains could be due to either an increase in gene expression or to redistribution of the proteins. To determine the expression levels of *grnd* and *egr*, we undertook quantitative polimerase chain reaction (qPCR) experiments with RNA extracted from control and GB larvae brains, which revealed a twofold increase of *egr* transcription in GB brains compared to controls (Fig. 2L) consistent with the increased Egr signal in glioma brains, and no significant differences for *grnd* transcription.

### Egr expressed in healthy tissue activates JNK in GB

**Fig. 3.**
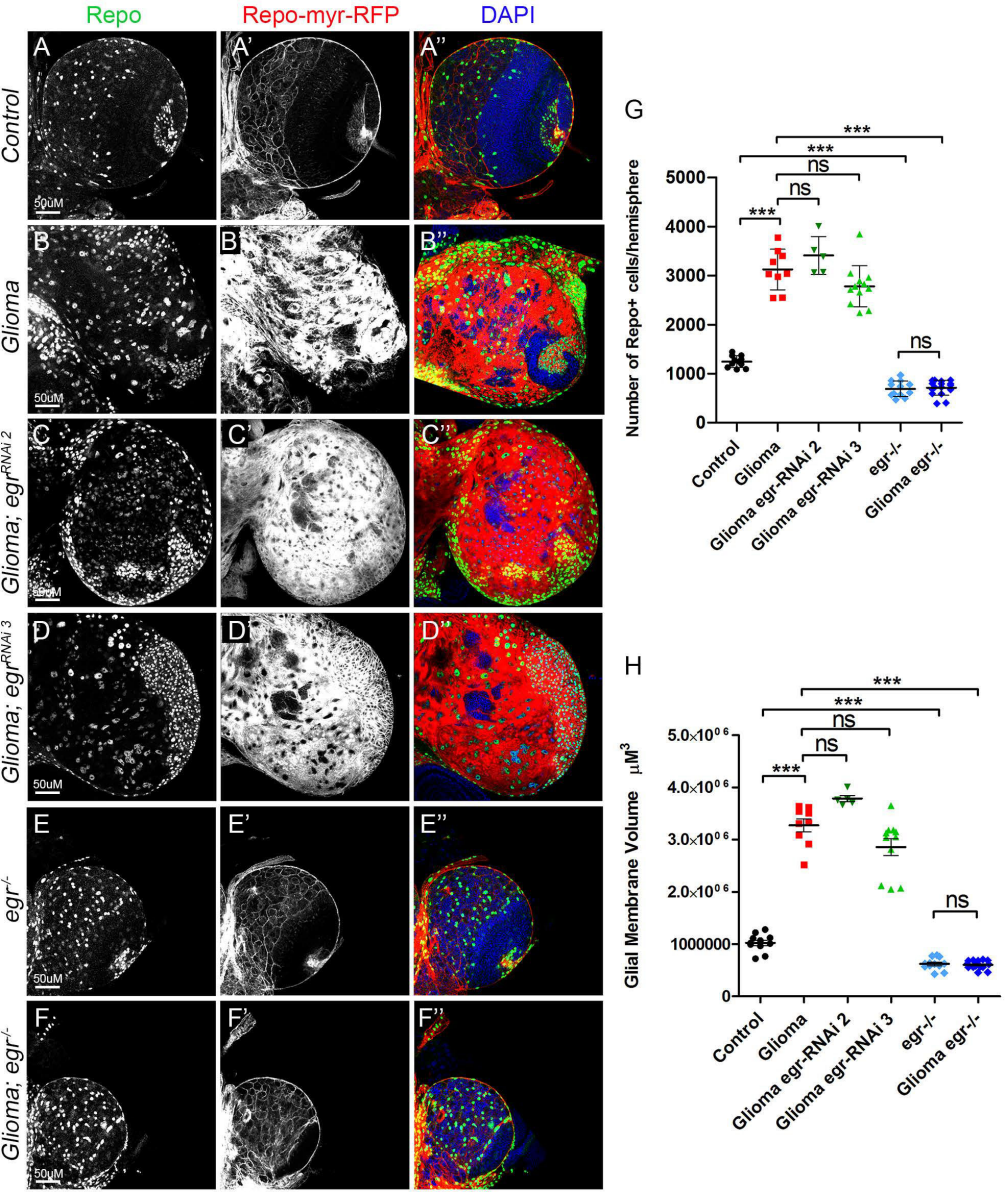
**Egr expressed in healthy tissue activates JNK in GB.** (A–F) Larval brain sections with glial membrane projections labelled in gray (red in the merge) and glial cell nuclei stained with Repo (gray, green in the merge). (A,B) GB brains show an increase in the number of glial cells and the volume of the TM network compared to control brains. (C,D) *egr* knockdown specifically in GB cells (using two different *egr-RNAi* lines) did not prevent GB cell number increase nor GB TM volume expansion. (E,F) Whole brain *egr* knockout in both GB cells and surrounding healthy tissue (*egr^−/−^*) prevented GB cell number increase and TM volume expansion. (G,H) The number of Repo+ cells is quantified in panel (G) and glial/glioma network volumes are quantified in (H). Nuclei are marked with DAPI (blue). Scale bar size is indicated in all figures. *n*=2 independent experiments, *n*>5 samples analysed for each genotype per experiment. One-way ANOVA with Bonferroni post-hoc test. Error bars show mean±s.d.; ***P<0.0001 or ns for non-significant. The expression system was active, and the GB induced, during the whole development including both embryonic and larval stages in all experiments in this figure. Genotypes: (A) *repo-Gal4, myr-RFP/UAS-lacZ;* (B) *UAS-dEGFR^λ^, UAS-dp110^CAAX^;; repo-Gal4, myr-RFP;* (C) *UAS-dEGFR^λ^, UAS-dp110^CAAX^;; UAS-egr-RNAi; repo-Gal4, myr-RFP;* (D) *UAS-dEGFR^λ^, UAS-dp110^CAAX^;; repo-Gal4, myr-RFP/UAS-egr-RNAi;* (E) *egr-/egr-; repo-Gal4, myr-RFP;* (F) *UAS-dEGFR^λ^, UAS-dp110^CAAX^; egr^−^/egr^−^; repo-Gal4, myr-RFP*.

The qPCR results that show transcriptional upregulation of *egr* in GB brains were obtained from whole brains extracts, therefore we cannot determine which specific cells upregulate *egr*. To determine whether the source of Egr is the glia, we silenced *egr* expression specifically in glioma cells. We used two different RNAi lines to knockdown *egr* (*egr-RNAi*) in GB cells (*repo>dp110^CAAX^*; *EGFR^λ^*; *egr-RNAi*). These two *UAS-egr-RNAi* lines have been previously validated, *egr-RNAi* 2 (Perkins et al., 2015) and *egr-RNAi* 3 (Beira et al., 2018; Doupé et al., 2018; Igaki et al., 2002). The quantifications show that *egr* knockdown in GB cells, does not prevent GB cell number increase (Fig. 3A–D and E) nor GB TMs expansion (Fig. 3A–D and F). Next we used a somatic mutant allele in homozygosis, egr^[MI15372]^ (Nagarkar-Jaiswal et al., 2015; Portela et al., 2019), to knockout *egr* expression in the whole animal, including GB cells and surrounding healthy tissue (denoted *egr^−/−^*). The analysis of confocal images indicates that the elimination of Egr prevents GB cell number increase (Fig. 3E–G) and TM volume expansion (Fig. 3F and H).

Together, our results show an increase of Egr protein in GB TMs (Fig. 2), however, *egr* expression in GB cells is not relevant for GB progression. Nevertheless, *egr* knockout prevents GB progression. Consistently, *egr* knockout phenocopies the effects of blocking JNK pathway signalling, via *grnd* knockdown or by expressing a dominant negative form of the effector Bsk (*BskDN*) (Adachi-Yamada et al., 1999) in GB cells.

Next, to determine if Egr produced in neurons is capable of activating the JNK pathway in neighbouring glial cells, we overexpressed *egr* under the control of the specific neuronal lineage driver *dnab-Gal4* (Fig. S2B) and monitored JNK activity with a standard JNK reporter [*puckered* (*puc*)*-LacZ*]. This construct monitors the transcriptional activation of the downstream JNK target *puckered* (Langen et al., 2013; Martin-Blanco et al., 1998). The confocal images of brain samples show neuronal lineages labelled with CD8-GFP (green) and the neuron-specific marker anti-ELAV (red), and intercalated glial cells stained with anti-Repo (blue) (Fig. S2B). Under normal conditions, (Fig. S2C), *puc-LacZ* reporter revealed that the JNK pathway activity is moderated in brain cells. However, upon *egr* overexpression in neurons, the JNK pathway reporter is activated in both neurons and in the surrounding glial cells (Fig. S2D,E). These results indicate that *egr* overexpressed in neurons can activate the JNK pathway in the surrounding glial cells.

Thus, we propose that the surrounding healthy brain tissue is the source of Egr, which in a GB condition progressively relocates to the GB TMs and activate JNK signalling through Grnd.

### Progressive JNK activation in glioma

The JNK pathway is upregulated in a number of tumours including GB and it is related to glioma malignancy (Hagemann et al., 2005; Huang et al., 2003; Mu et al., 2018; Zeng et al., 2018). Moreover, JNK is a target for specific drugs in combination with TMZ treatments as it was proven to play a central role in GB progression (Feng et al., 2016; Kitanaka et al., 2013; Matsuda et al., 2012; Okada et al., 2014). However, little is known about the molecular mechanisms underlying JNK activation in GB cells and the functional consequences for GB progression.

We have previously shown that the JNK pathway is activated in *Drosophila* GB cells (Portela et al., 2019), by using the *TRE-RFP* reporter that confers transcriptional activation in response to JNK signalling (Chatterjee and Bohmann, 2012; Jemc et al., 2012; Ruan et al., 2016). To study the temporal activation of the JNK pathway, and to uncover the order of the signalling events in GB, we took advantage of another standard JNK reporter, *puc-LacZ*. We chose to use this reporter because it is more specific and it is activated earlier than the *TRE-RFP* reporter (Langen et al., 2013; Martin-Blanco et al., 1998). To control the temporal induction of the tumour, we used the thermo sensitive repression system Gal80^TS^. Individuals maintained at 17°C do not activate the expression of the UAS constructs, after larvae were switched to 29°C, the protein Gal80^TS^ was not longer able to repress Gal4 system and the genetic induction of GB was activated. In addition, GB cells were marked with RFP (*UAS-myr-RFP*) to distinguish GB cells and healthy tissue.

**Fig. 4.**
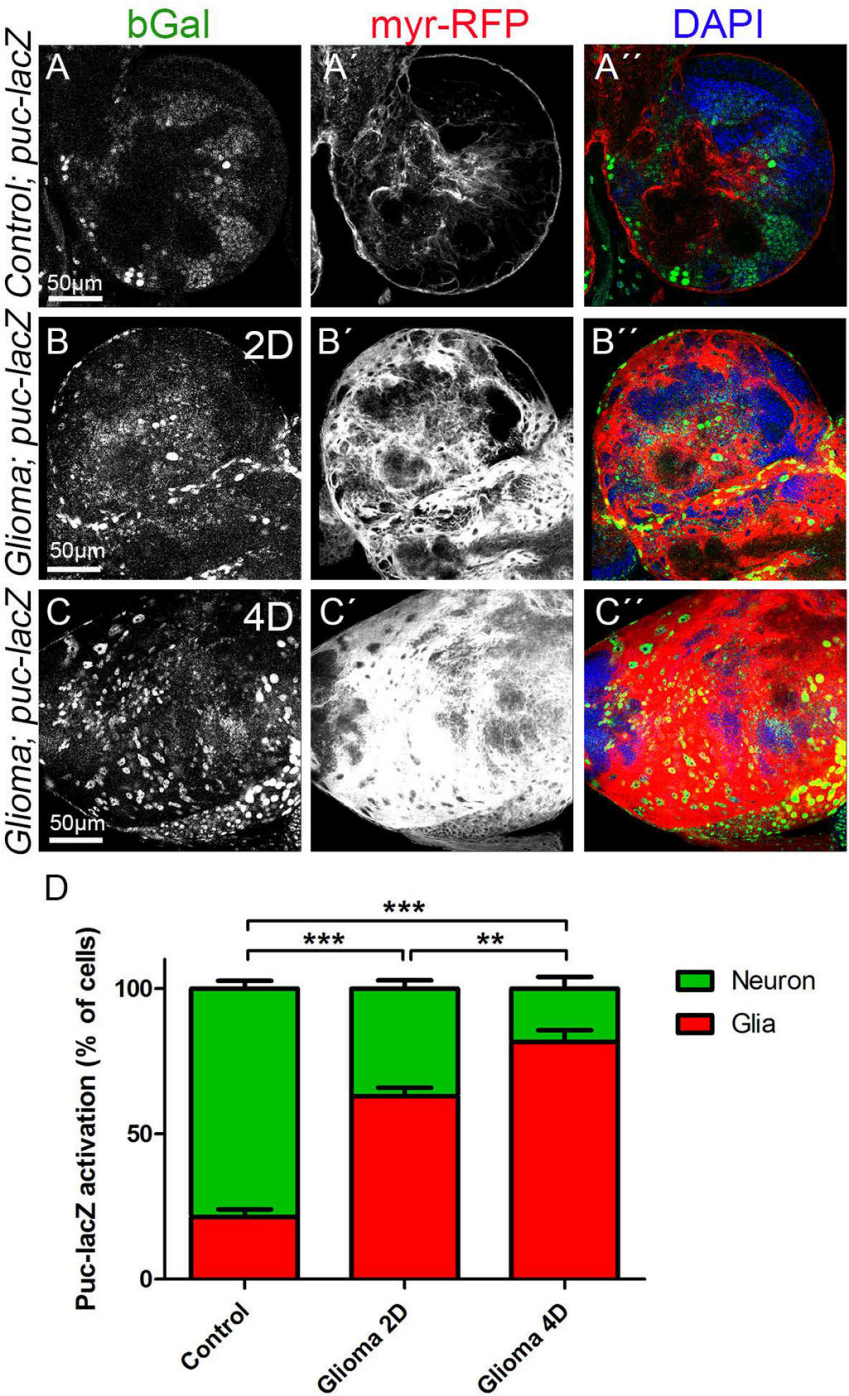
**JNK signalling pathway activation in GB.** Larval brain sections from third-instar larvae displayed at the same scale. Glial cell bodies and membranes are labelled with *UAS-myr-RFP* (gray or red in the merge) driven by *repo-Gal4* (A–C) JNK signalling pathway reporter *puc-lacZ* (stained with anti-bGal, gray or green in the merge) in (A) control, (B) glioma induced for 2 days (2D) and (C) glioma induced for 4 days (4D). (A) *puc-lacZ* reporter signal revealed by bGal staining shows activation of the JNK pathway throughout the brain. 22% of the bGal signal localised in glial cells and most of the signal (78%) localised in the surrounding tissue in control brains. (B,C) *puc-lacZ* reporter bGal signal shows a progressive activation in GB cells, from 63% after 2 days of GB induction (B) to 80% after 4 days of GB induction (C). (D) Quantification of the % of cells with *puc-lacZ* activation in glial cells and in the surrounding tissue. Nuclei are marked with DAPI (blue). *n*=2 independent experiments, *n*=8 samples analysed for each genotype per experiment. One-way ANOVA with Bonferroni post-hoc test. Error bars show mean±s.d.; **P<0.001, ***P<0.0001. Scale bar size is indicated in all figures. The expression system was active, and the GB induced, during the whole development including both embryonic and larval stages in panel A. In panels B and C animals were raised at 17°C, shifted to 29°C for 2 or 4 days at ∼6 or 2 days after egg laying (AEL), respectively. Genotypes: (A) *Gal80^ts^/repo-Gal4, myr-RFP/puc-lacZ;* (B,C) *UAS-dEGFR^λ^, UAS-dp110^CAAX^; Gal80^ts^; repo-Gal4, myr-RFP/puc-lacZ*.

We analysed JNK activity in GB at two time points, 2 and 4 days after GB induction. *Puc-LacZ* reporter signal revealed by anti-bGal staining shows activation of the JNK pathway throughout the brain. We quantified the b-Galactosidase (*puc-LacZ*) signal that overlaps with the glial population (RFP). The analysis of confocal images showed that 22% of the bGal signal localised in glial cells and *puc-LacZ* is mostly activated in the tissue surrounding the glial population (∼78%) in control brains (Fig. 4A). 2 days after GB induction, *puc-LacZ* activation in healthy tissue surrounding GB cells is reduced (∼37%) and GB cells show a progressive activation of the JNK reporter: from 63% of *puc-LacZ* signal in GB (2 days), to ∼80% (4 days) (Fig. 4B–D). These results suggest that the JNK pathway is progressively activated in GB cells as the tumour progresses.

### Timeline: GB first causes neurodegeneration, and then TMs infiltration and cell number increase

**Fig. 5.**
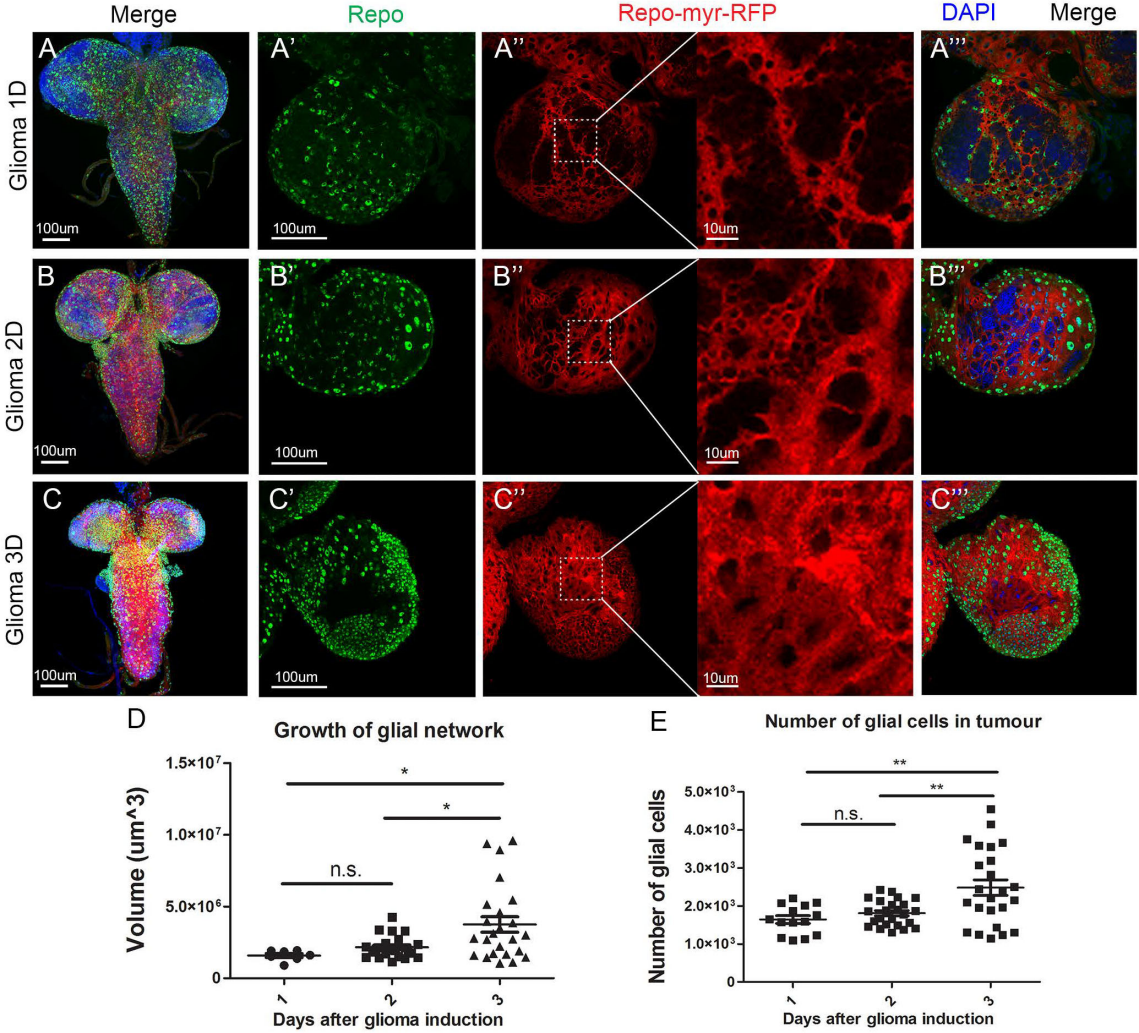
**GB TM network volume and number of glial cells after 1, 2 and 3 days of tumour induction.** Larval brain sections from third-instar larvae at several magnifications (A–C) show whole brains, (A′–C‴) show single brain hemispheres. Glial cell bodies and membranes are labelled with *UAS-myr-RFP* (red) driven by *repo-Gal4* and glial cell nuclei are stained with Repo (green) and visualised as green spots. The insets of the red channel in (A″–C″) show higher magnifications for a detailed visualisation of the glioma network of TMs. (A) Third-instar larval brain after 1 day of tumour induction. (B) Third-instar larval brain after 2 days of tumour induction. (C) Third-instar larval brain after 3 days of tumour induction. (D) Quantification of the/glioma network volumes after induction of the glioma for 1, 2 or 3 days. (E) Quantification of the number of Repo+ cells after induction of the glioma for 1, 2 or 3 days. Nuclei are marked with DAPI (blue). Scale bar size is indicated in all figures. *n*=3 independent experiments, *n*>10 samples analysed for each timepoint per experiment. One-way ANOVA with Bonferroni post-hoc test. Error bars show mean±s.d.; *P≤0.05; **P≤0.01; n.s., not significant. Animals were raised at 17° C, shifted to 29°C for 1, 2 or 3 days at ∼8, 6 or 4 days AEL, respectively. Genotypes: (A-C) *UAS-dEGFR^λ^, UAS-dp110^CAAX^; Gal80^ts^; repo-Gal4, myr-RFP*.

The previous results indicate that JNK signalling pathway activation is progressive, and therefore GB progression follows a chronogram. To evaluate the cellular events associated with GB (progressive growth of the glial membrane projections and the number of glial cells); we dissected larval brains at 1, 2 and 3 days after tumour induction (referred to as 1D, 2D or 3D in Fig. 5A–C). GB TMs were visualised with *UAS-myr-RFP* and the glia nuclei were stained with a specific anti-Repo antibody to quantify the total glial cell number (Fig. 5A–C).

The statistical analysis of glial membrane volume quantifications (Fig. 5D) shows no significant differences in the volume of the network between 1 day and 2 days of tumour induction. Nevertheless, there is a significant increase in the volume of the TMs between 2 and 3 days of tumour induction. Similarly, the statistical analysis of the number of glial cells (Fig. 5E) shows no significant increase in the number of glial cells between day 1 and day 2, but there is a significant increase between day 2 and day 3, and between day 1 and day 3 after tumour induction. This suggests a progressive growth and expansion of the glial TMs and a progressive increase in the number of GB cells.

To evaluate the impact of the progressive GB growth and membrane expansion on the surrounding cells, we studied synapses in the motor neurons. In the *Drosophila* larvae, motor neurons are localised in the CNS and project their axons towards the neuromuscular junction (NMJ), a well-established system to evaluate neurodegeneration (Keshishian et al., 1996; Portela et al., 2019). A great advantage of these stereotyped neurons is the possibility of counting, rather than estimating, the number of synapses. We are focused on the early stages of neurodegeneration, hence the interest in documenting synapse loss rather than neuronal death, which is a later event in a neurodegenerative process.

**Fig. 6.**
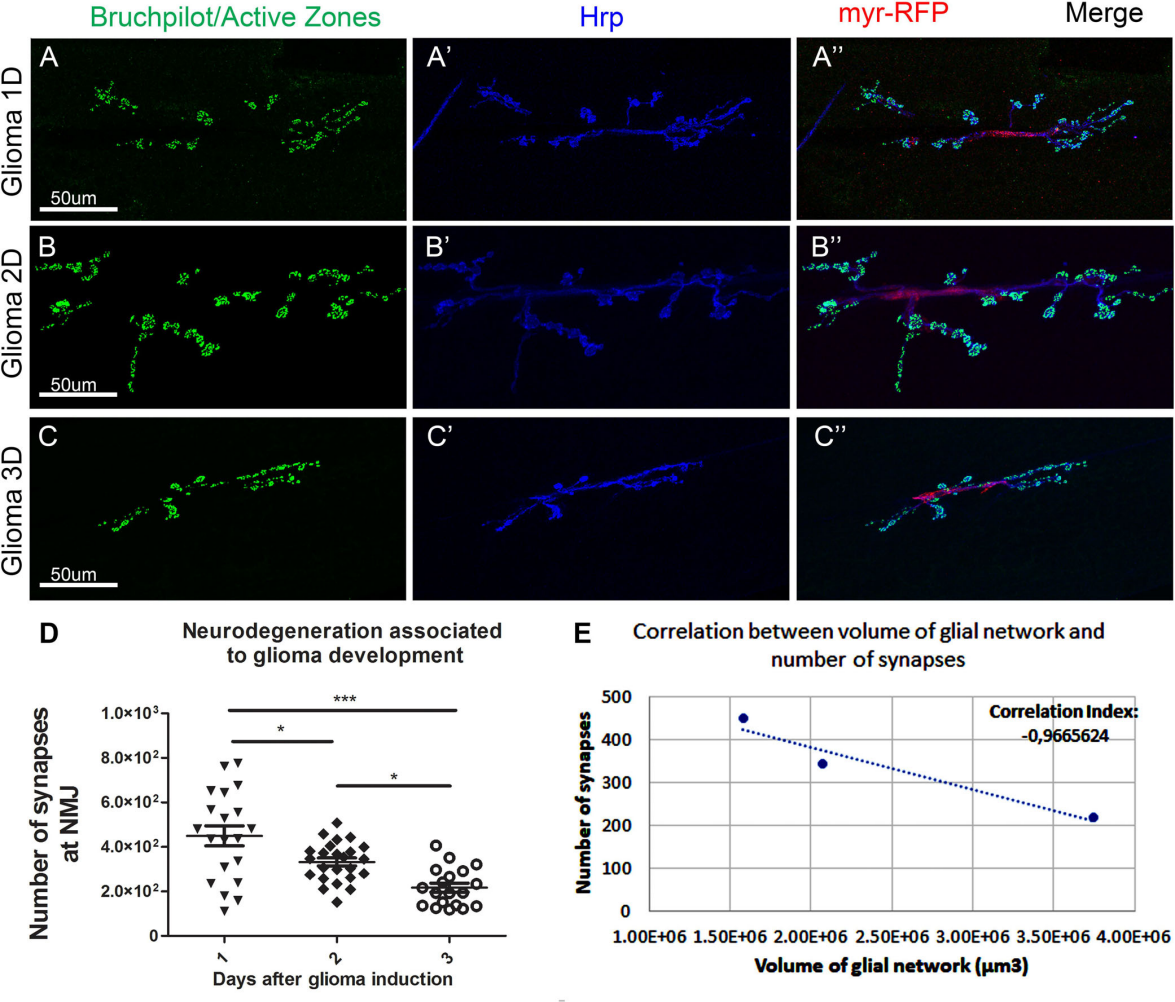
**Number of synapses at the NMJ of third-instar larvae after 1, 2 and 3 days of tumour induction.** Neurons from the larval NMJ stained with Bruchpilot (NC82) show the synaptic active zones. The synaptic connexions at the NMJ of *Drosophila* larvae are between muscles six and seven in the third and fourth abdominal segments. Anti-Bruchpilot (α-NC82) binds to Bruchpilot marking presynaptic zones in green. Anti-Hrp marks the neuron membrane in blue. Glial cell bodies and membranes are labelled with *UAS-myr-RFP* (red) driven by *repo* Gal4. (A) Third-instar larval NMJ after 1 day of tumour induction. (B) Third-instar larval NMJ after 2 days of tumour induction. (C) Third-instar larval NMJ after 3 days of tumour induction. (D) Graphical representation of the number of synapses at the NMJ after 1, 2 or 3 days of GB induction. Scale bar size is indicated in all figures. *n*=3 independent experiments, *n*>10 samples analysed for each timepoint per experiment. One-way ANOVA with Bonferroni post-hoc test. Error bars show mean±s.d.; ***P≤0.001; * *P*≤0.05. (E) Scatter plot showing the correlation between volume of the TMs network from Fig. 5D and number of synapses at the NMJ (Fig. 6D). There is a negative correlation between the volume of the network and the number of synapses, therefore, at smaller volumes of glial network there are more synaptic connexions at the NMJ and as the TM network volume increases, more synapses are lost. Animals were raised at 17°C, shifted to 29°C for 1, 2 or 3 days at ∼8, 6 or 4 days AEL, respectively. Genotypes: (A–C) *UAS-dEGFR^λ^, UAS-dp110^CAAX^; Gal80^ts^; repo-Gal4, myr-RFP*.

We quantified the number of synapses in the NMJ of third-instar larvae after 1, 2 and 3 days of GB induction. NMJs were stained with anti-bruchpilot to visualise active zones (synapses) (Fig. 6A–C). The statistical analysis of synapse number (Fig. 6D) show progressive synapse loss between 1 and 2 days, and between 2 and 3 days of tumour induction. The overall loss of synapses at the NMJ is highly significant (Fig. 6D).

To determine whether there is an association between the volume of the glial network and the number of synapses at the NMJ, we plotted the number of synapses against the volume of the glial network in a correlation graph (Fig. 6E). The correlation index is −0.966 (3 s.f.) indicating a negative association between the volume of the TMs and the number of synapses at the NMJ. Therefore, larger glial TMs network correlates with a greater synapse loss.

Our previous results indicate that GB cells increase the physical interaction with neuronal membranes (Portela et al., 2019), this cellular interaction is required for neuronal Wg depletion by GB, which causes synapse loss. Moreover, the prevention of TMs expansion or Wg depletion prevents GB cell number increase, GB progression and synapse loss. In addition, our new results show that neurodegeneration and GB volume expansion occurs prior to cell number increase. Cell-to-cell communication therefore gains a particular relevance for GB development and progression.

**Fig. 7.**
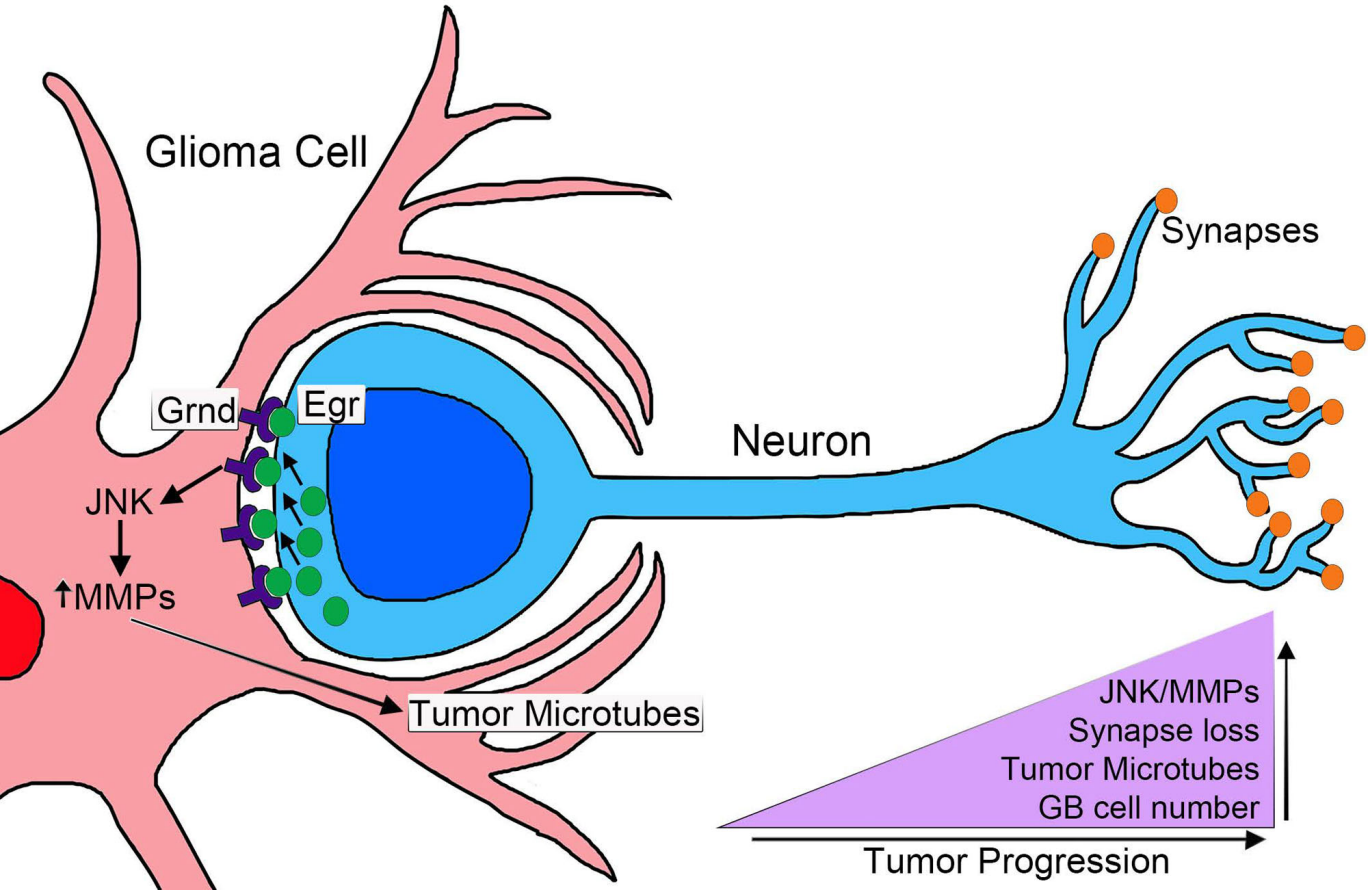
**Cell to cell communication mediate GB progression in *Drosophila.*** Glioma cells produce a network of TMs that grow to reach the surrounding tissue that includes neurons. Grnd is increased in the GB TMs and Egr is produced in the surrounding tissue. Egr activates the JNK signalling pathway through Grnd in GB. As a consequence, MMPs are upregulated in GB and facilitate further TM infiltration in the brain that is necessary for synapse loss in the surrounding neurons and GB cell number increase. This results in tumour progression and lethality.

Taking all these data together, we propose that the communication between GB cells and neurons is relevant for this disease, and must play a role in the initial steps of GB before cell proliferation (Fig. 7). Therefore, these results bring the study of cell-to-cell communication as a relevant mechanism for GB.

## DISCUSSION

Activating mutations in EGFR and PI3K pathways are the most frequent initial signals in GB. However, the attempts to treat GB reducing the activation of these pathways have so far been limited. GB cells show a high mutation rate and usually present several sub-clones within the same patient and from the same primary tumour (McGranahan and Swanton, 2017; Qazi et al., 2017). The current literature suggest that a multiple approach is required to obtain better results (Prasad et al., 2011; Taylor et al., 2012; Westhoff et al., 2014; Westphal et al., 2017; Zhao et al., 2017), and we propose that the communication between GB and the surrounding healthy tissue is a central mediator of tumour progression.

### Progressive tumour growth

Although there is an overall significant expansion in volume of the glial TMs membrane and an increase in the number of glial cells in the *Drosophila* model of GB, there is no early significant increase in those parameters. Tumour volume shows values above the average after 2 days of tumour induction, and a great dispersion in the volume of the network and the number of glial cells after 3 days of tumour induction. Previous publications show that under physiological conditions, there is a stable state that prevents cells from exiting the cell cycle, dividing or activating the actin cytoskeleton to extend further a network of membrane projections (Portela et al., 2019; Read et al., 2009). Therefore, after 1 day of tumour induction, GB cells do not show evident changes, there is a uniform range of values for the volume of membrane projections and number of glial cells. After 2 days of tumour induction, some individuals abandon this stable state and GB cells extend larger and longer membrane projections, although most individuals continue in stability and display morphological characteristics similar to control samples. However, after 3 days of tumour induction there is a great expansion of membrane projections and the number of glial cells is increased. Similar to GB patients, some individuals are more resistant than others to the loss of homeostasis, which would explain the variance in the values for network volume and number of glial cells after 3 days of tumour induction.

### Progressive neurodegeneration

There is a significant progressive decrease in the number of synapses at the NMJ. Nevertheless, the variance in the number of synapses after 1 day of tumour induction is very large, reaching uniform values after 2 and 3 days of tumour induction. The individuals show different resistances to the changes caused by the GB. After 1 day of tumour induction, some individuals are significantly affected by the tumour and suffer a great loss of synapses at the NMJ while other individuals are more resistant, and the number of synapses remains normal. After 2 days of tumour induction, the impact of GB causes severe changes to all individuals regardless of their initial resistance.

We have observed a negative correlation between the volume of the membrane network and the number of synapses at the NMJ, suggesting that the expansion of the membrane projections is responsible for the neurodegeneration. Previous studies from our laboratory have proved that TMs surround neurons and deplete WNT from them (Portela et al., 2019), which leads to synapse loss and neurodegeneration (Rich and Bigner, 2004). We can conclude that as the tumour progresses, TMs grow progressively, infiltrate in the brain and surround neurons leading to their degeneration, the process previously described as vampirization (Portela et al., 2019). The three main events described here do not occur concomitantly, the progressive neurodegeneration is an earlier event in GB development, associated to early neuronal Wg/WNT depletion by GB cells. The expansion of the membrane network of TMs is slow during the first 24 h of tumour development but increases after 48 h. TMs expansion and infiltration require Wg/WNT and JNK/MMPs pathways activation. Consequently, TMs expansions facilitate GB cells to further contact and communicate with the surrounding tissue. Eventually, Wg/WNT and JNK signals promote the increase in the number of GB cells that occurs as a later event. These evidences indicate that GB-neuron interaction is an earlier event required for GB progression. Consistent with this, a recent study in *Drosophila*, showed inter-tissue communication between epithelial tumoural cells and the adjacent healthy mesenchymal cells. The tumoural epithelial cells upregulate and transport the Notch ligand Delta in cytonemes, which contact and communicate with the healthy mesenchymal cells to activate Notch signalling in a non-autonomous manner. Notch activity keep the mesenchymal cell precursors in an undifferentiated and proliferative state that is critical to sustain tumour progression (Boukhatmi et al., 2020). This cell to cell interaction goes in line with our findings in glia–neuron communication in GB, where glial cells use cytonemes/TMs to retrieve Egr from the surrounding healthy tissue and activate JNK signalling in the tumour cells to sustain tumour progression.

### Progressive activation of JNK pathway via Egr/Grnd

In this manuscript, we expand our knowledge regarding cell to cell communication to JNK signalling pathway. One of the main molecular events in GB is the activation of JNK signalling that regulates MMPs expression, TMs expansion and infiltration. Previous results indicate that JNK pathway responds to Wg and TMs network expansion. We described these three events as a regulatory positive feedback loop in GB progression (Portela et al., 2019). However, the signals that specifically activate JNK pathway are under debate. Grnd is a receptor that upon binding to the ligand Egr activates JNK pathway (Fig. 7). The qPCR results and the localisation analysis of Egr-GFP fused protein suggest that Egr is produced in the healthy surrounding tissue. The genetic knockdown of *egr* in GB cells does not rescue cellular features such as the TM network volume and GB cell number, or the lethality caused by the GB. However, when a GB is induced in an *egr* somatic mutant background (*egr−/−*) the tumour does not grow (Fig. 3E,F). Therefore, if *egr* expression in GB cells is not relevant for GB, but *egr* is upregulated in GB brain samples and *egr* knockout prevents TM network expansion and GB cell number increase, these data suggest that the main source of Egr is not the GB but the surrounding healthy tissue. However, further analysis will be required to investigate the contribution of autonomous Egr production to the morphology of the GB expansion. Under physiological conditions, a fraction of Egr is found in glial cells. However, under GB conditions there is an increase of Egr accumulated in the membrane of GB cells in contact with healthy neuronal tissue. The progressive activation of JNK pathway in glial cells correlates with the morphological changes (membrane network expansion and number of glial cells) that GB cells undergo after 2 days of tumour induction. Therefore, this example of neuron-GB interaction where non-autonomous signals facilitate tumour progression and GB-mediated neurodegeneration, contributes to the complexity and versatility of these incurable tumours and highlights the relevance of the interactions and signalling between GB and the surrounding healthy tissue (Fig. 7). Therefore, it is of interest to uncover the regulatory mechanisms that mediate *egr* expression and secretion in neurons in response to GB induction, and the associated response in GB cells mediated by Grnd/Egr as a potential modulator of GB progression.

## MATERIALS AND METHODS

### Fly stocks

Flies were raised in standard fly food at 25°C. Fly stocks from the Bloomington stock Centre: *UAS-lacZ* (BL8529)*, UAS-myr-RFP* (BL7119), *repo-Gal4* (BL7415), *tub-gal80^ts^* (BL7019), *Egr-GFP* (BL66381), *UAS-egr-RNAi* 2 (BL 55276) and *UAS-egr-RNAi* 3 (BL58993), egr^[MI15372]^ (BL59754), *dnab-Gal4, UAS-CD8-GFP* (gift from L. Y. Cheng), *UAS-dEGFR^λ^*, *UAS-PI3K92E* (*dp110^CAAX^*) (gift from R. Read), *UAS-ihog-RFP* (gift from I. Guerrero), *puc-lacZ* (gift from E. Martín-Blanco), *UAS-dmyc* (Moreno and Basler, 2004), *UAS-TOR-DER^CA^* (Domínguez et al., 1998).

### *Drosophila* glioblastoma model

In *Drosophila*, a combination of EGFR and PI3K mutations effectively causes a glioma-like condition that shows features of human gliomas including glia expansion, brain invasion, neuron dysfunction, synapse loss and neurodegeneration (Kegelman et al., 2014; Portela et al., 2019; Read, 2011; Read et al., 2009). To generate a glioma in *Drosophila* melanogaster, the Gal4/UAS system (Brand and Perrimon, 1993) was used as described above (*repo*-Gal4>UAS-*EGFR^λ^*, UAS-*dp110^CAAX^*). The expression system was active during the whole development including both embryonic and larval stages in all experiments in Figs 1–3 and Fig. S1. To restrict the expression of this genetic combination and control it in a temporal manner, we used the thermo-sensitive repression system Gal80^TS^. Individuals maintained at 17°C did not activate the expression of the UAS constructs, but when the larvae were switched to 29°C, the protein Gal80^TS^ changed conformation and was not longer able to bind to Gal4 to prevent its interaction with UAS sequences, and the expression system was activated and therefore the GB was induced. This system was used in all experiments in Figs 4–6. *Tub-GAL80^TS^*; *repo*-Gal4>UAS-*EGFR^λ^*, UAS-*dp110^CAAX^* animals were raised at 17°C, shifted to 29°C for 1, 2, 3 or 4 days at ∼8, 6, 4 or 2 days AEL, respectively, and subjected to dissection immediately after.

### Antibodies for immunofluorescence

Third-instar larval brains were dissected in phosphate-buffered saline (PBS), fixed in 4% formaldehyde for 30 min, washed in PBS+0.1 or 0.3% Triton X-100 (PBT), and blocked in PBT+5% BSA.

Antibodies used were as follows: mouse anti-Wg [Developmental Studies Hybridoma Bank (DSHB) 4D4 1:50 (Brook and Cohen, 1996)], mouse anti-Repo [DSHB 8D12 1:50 (Stork et al., 2014)], mouse anti-Fz1 [DSHB 1C11 1:50 (Park et al., 1994)], mouse anti-Cyt-Arm [DSHB N27A1 1:50 (Riggleman et al., 1990)], mouse anti-MMP1 [DSHB 5H7B11, 3A6B4, 3B8D12, 1:50 (Page-McCaw et al., 2003)], rabbit anti-MMP2 [1:500, (Dear et al., 2016)], guinea pig anti-Grnd [1:250 (Andersen et al., 2015)], mouse anti-β-galactosidase [Sigma-Aldrich G4644, 1:500 (Portela et al., 2019)], mouse anti-GFP [Invitrogen A11120, 1:500 (Djiane et al., 2005)], mouse anti-bruchpilot [DSHB nc82, 1:20 (Wagh et al., 2006)], Rabbit anti-Hrp [Jackson ImmunoResearch 111-035-144, 1:400 (Stork et al., 2014)].

Secondary antibodies: anti-mouse Alexa 488, 568, 647, anti-rabbit Alexa 488, 568, 647 (Thermo Fisher Scientific, 1:500). DNA was stained with 2-(4-amidinophenyl)-1H-indole-6-carboxamidine (DAPI, 1 µM).

### qRT-PCRs

Total RNA was isolated from larvae brains (Trizol, Invitrogen), and cDNAs were synthesised with M-MLV RT (Invitrogen).

qRT-PCR was performed using a 7500 Real Time PCR System (Applied Biosystems) with cycling conditions of 95°C for 10 min and 40 cycles of 95°C for 15 s and 55°C for 1 min. Each experimental point was performed with samples from two independent crosses and three replicates per experimental point. Results were normalised using the housekeeping Rp49 and the ΔΔ cycle threshold method and are expressed as the relative change (-fold) of the stimulated group over the control group, which was used as a calibrator.

qRT-PCR results were analysed with 7500 version 2.0.6 software (Applied Biosystems). *grnd* and *egr* qRT-PCRs were performed using Sybergreen (Applied Biosystems) with the following primers:

grnd F 5′-CAATGTGGCCCTGAAAACTT

grnd R 5′-TGAATTGGTTTTCCCCCATA

egr F 5′-ACTCCATTCCTGCAGTGCTT

egr R 5′-CCGGGGATAATCTCTCCAAT

### Imaging

Fluorescent labelled samples were mounted in Vectashield mounting media with DAPI (Vector Laboratories) and analysed by confocal microscopy (LEICA TCS SP5). In all experiments the whole brain lobes were acquired individually and whole stacks were analysed. The images shown in the figures are a single plane images to facilitate visualisation. In some panels, higher magnifications of single plane images are shown in the figures to facilitate the visualisation of the antibody stainings in more detail. Images were processed using Leica LAS AF Lite and Fiji (Image J 1.50e). Images were assembled using Adobe Photoshop CS5.1.

### Quantifications

Relative MMP1 and Grnd staining within brains was determined from images taken at the same confocal settings. Average pixel intensity was measured using measurement log tool from Fiji 1.51g and Adobe Photoshop CS5.1. Average pixel intensity was measured in the glial tissue and in the adjacent healthy tissue and expressed as a ratio. Glial network volume was quantified using Imaris surface tool (Imaris 6.3.1 Bitplane Scientific Solutions software).

The number of Repo^+^ cells, the number of synaptic active sites and the number of puc-lacZ positive cells was quantified by using the spots tool Imaris 6.3.1 software, we selected a minimum size and threshold for the puncta in the control samples of each experiment. Then we applied these conditions to the analysis of each corresponding experimental sample. For the puc-lacZ glia or healthy surrounding tissue co-localisation studies we quantified the total number of puc-lacZ^+^ cells and then applied a co-localisation filter (intensity centre of the channel of interest) using the Spots tool from the Imaris 6.3.1 software.

For the co-localisation of Egr-GFP in glial cells, GFP channel volume was quantified using Imaris surface tool. We selected a specific threshold for the total volume in the control samples and then we applied these conditions to the analysis of the corresponding experimental sample. Then we applied a co-localisation filter (intensity mean of the red channel).

### Statistical analysis

To analyse and plot the data, we used GraphPad Prism 6. We performed a D'Agostino & Pearson normality test and the data found to have a normal distribution were analysed by a two-tailed *t*-test with Welch-correction. In the case of multiple comparisons, we used a one-way ANOVA with Bonferroni post-hoc test. The data that did not pass the normality test were subjected to a two-tailed Mann–Whitney *U-*test or in the case of multiple comparisons a Kruskal–Wallis test with Dunn’s post-hoc test. Error bars represent standard error of the mean. * represents *P*-value≤0.05; **P-value≤0.01; ***P-value≤0.001. Statistical values of *P*-value >0.05 were not considered significant, (n.s.).

## Supplementary Material

Supplementary information
