# Supplementary material for: Cell-to-cell communication mediates glioblastoma progression in Drosophila
Source: Biol Open. 2020 Sep 29;9(9):bio053405. doi: 10.1242/bio.053405 (PMC7541342; doi:10.1242/bio.053405)
Supplement: Supplementary information [file biolopen-9-053405-s1.pdf]

Figure S1

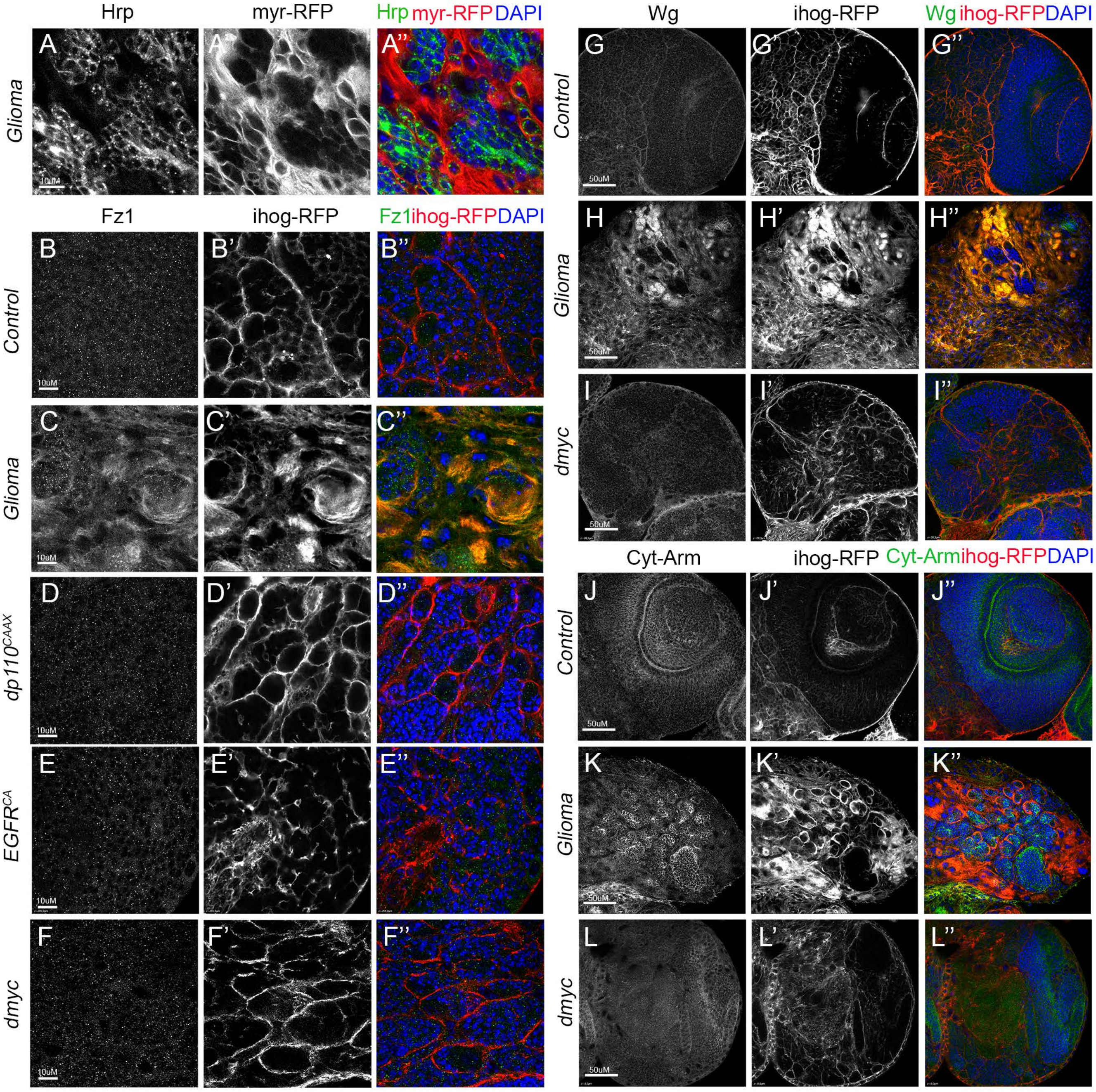

Biology Open • Supplementary information

Figure S1: Independent constitutive activation of PI3K or EGFR or ectopic *dmyc* are not responsible for Wg/Fz1 accumulation and expansion of TM network.

Brain sections from third instar larvae. (A) Glia is labeled with *UAS-myr-RFP* (gray or red in the merge) driven by *repo-Gal4* to visualize glial cell bodies and membranes and neurons are stained with Hrp (gray or green in the merge) and enwrapped by glial cell membranes in glioma brains (merge). (B-F) Glia are labeled with *UAS-Ihog-RFP* (gray or red in the merge) driven by *repo-Gal4* to visualize active cytonemes/ TM structures in glial cells and stained with Fz1/Wg or Cyt-Arm (gray or green in the merge). (B-F) Fz1 (gray or green in the merge) is homogeneously distributed in control brain sections, with a slight accumulation in the cytonemes. (B). (C) Fz1 accumulates in the TMs in glioma brains. Fz1 is homogeneously distributed in (D) *dp110*^{CAAX}, (E) *EGFR*^{CA} and (F) *dmyc* brain sections similar to the control (B). (G-L) Wg and Cyt-Arm (green) are homogeneously distributed in control brain sections (G, J) as well as in *dmyc* sections (I, L) and the TMs network revealed by Ihog-RFP (gray or red in the merge) remains similar to control brain sections and do not enwrap neuronal clusters as opposed to GB brains where Wg accumulates in TMs (H) and Cyt-Arm accumulates in the neurons' cytoplasm where it is inactive (K). Nuclei are marked with DAPI (blue). The expression system was active, and the GB induced, during the whole development including both embryonic and larval stages in all experiments in this figure.

Genotypes:

(A) *UAS-dEGFR*$^{\lambda}$*, UAS-dp110*^{CAAX}*; Gal80*^{ts}*; repo-Gal4, myr-RFP*

(B, G, J) *UAS-lacZ/repo-Gal4, UAS-ihog-RFP*

Biology Open • Supplementary information

(C, H, K) *UAS-dEGFR*$^{\lambda}$*, UAS-dp110*^{CAAX}*;; repo-Gal4, UAS-ihog-RFP*

(D) *UAS-dp110*^{CAAX}*;; repo-Gal4, UAS-ihog-RFP*

(E) *UAS-TOR-DER*^{CA} *;; repo-Gal4, UAS-ihog-RFP*

(F, I, L) *repo-Gal4, UAS-ihog-RFP/UAS-dmyc*

Figure S2

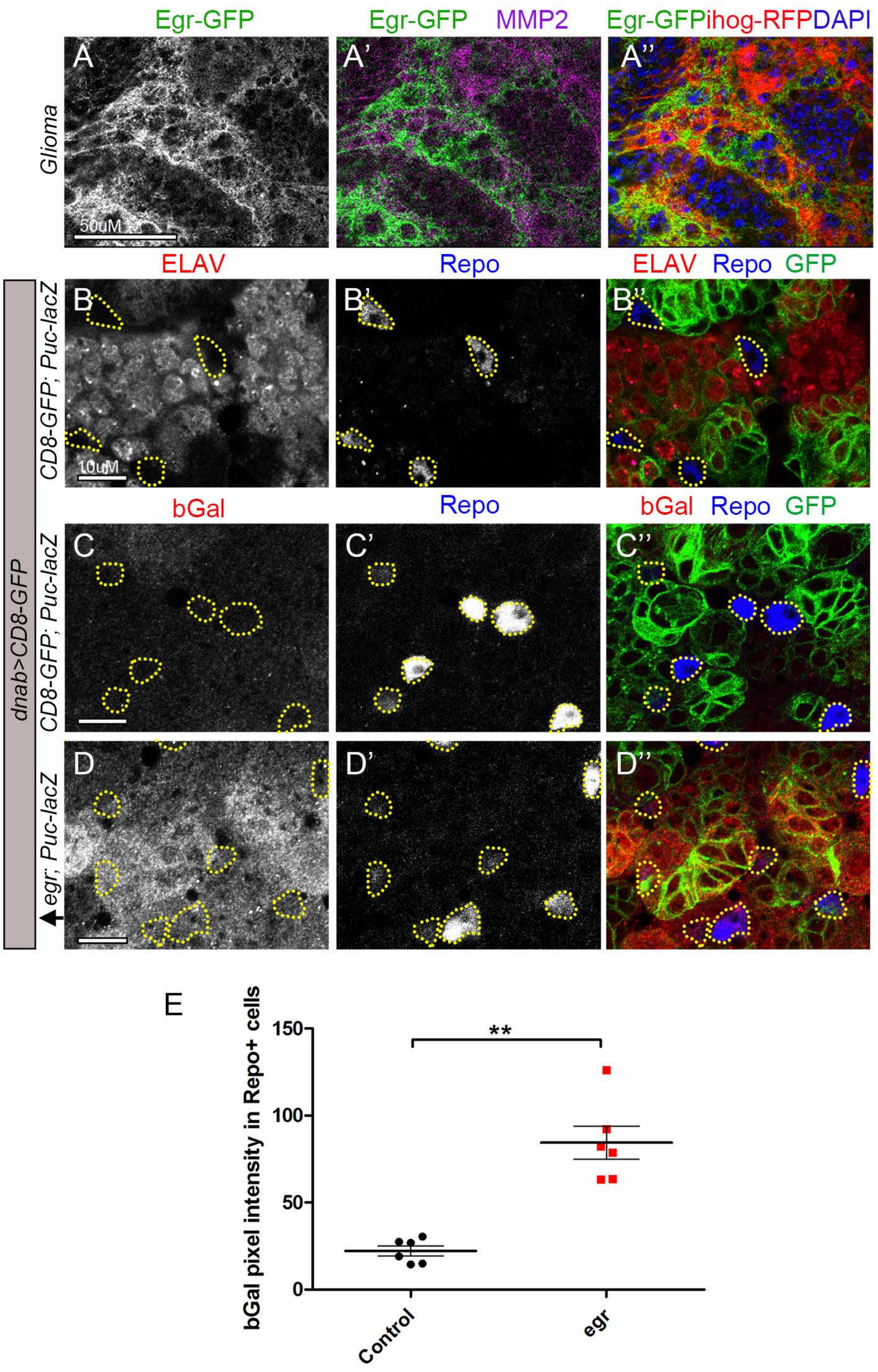
Egr-GFP
Egr-GFP MMP2
Egr-GFP ihog-RFP DAPI
A
A'
A''
Glioma
50uM
ELAV
Repo
ELAV Repo GFP
B
B'
B''
CD8-GFP; Puc-lacZ
10uM
bGal
Repo
bGal Repo GFP
C
C'
C''
dnab>CD8-GFP
CD8-GFP; Puc-lacZ
D
D'
D''
egr; Puc-lacZ
E
bGal pixel intensity in Repo+ cells
150
100
50
0
**
Control
egr

Figure S2: *egr* overexpressed in neurons can activate JNK pathway in the surrounding glial cells.

Brain sections from third instar larvae displayed at the same scale. Glia are labelled with *UAS-Ihog-RFP* (gray or red in the merge) driven by *repo-Gal4* to visualize TM structures in glial cells. Egr protein is visualized by GFP staining (green) of a transgenic *Drosophila* line in which the endogenous *egr* gene is GFP tagged (Egr-GFP protein fusion reporter) and stained with MMP2 (gray or magenta in the merge), Nuclei are marked with DAPI (blue). (A) MMP2 (gray or magenta in the merge) accumulates in the TMs and partially colocalizes with Egr-GFP (green) signal in GB brains. (B-D) Brain sections from third instar larvae displayed at the same scale. Neuronal lineages are labelled with *UAS-CD8-GFP* (green) driven by *dnab-Gal4* to visualize neuronal membranes. (B) Control brains show neurons labelled with anti-ELAV (red) and intercalated glial cells labelled with anti-Repo (blue). (C-D) JNK signaling pathway reporter *puc-lacZ* (stained with anti-bGal, gray or red in the merge) in (C) control brains show moderated *puc-lacZ* in brain cells. (D) *egr* overexpression in neurons show *puc-lacZ* activation in both neurons and in the surrounding glial cells. n=1 independent experiment, n=6 samples analyzed for each genotype. two-tailed t test with Welch correction. Error bars show mean±s.d.; ** P=0.0015. Scale bar size is indicated in all figures. The expression

Biology Open • Supplementary information

system was active during the whole development including both embryonic and larval stages in all experiments.

Genotypes:

(A) *UAS-dEGFR*$^{\lambda}$*, UAS-dp110*^{CAAX}*;Egr-GFP; repo-Gal4, UAS-ihog-RFP*

(B-C) *UAS-CD8-GFP ; dnab-Gal4, UAS-CD8-GFP/puc-lacZ*

(D) *UAS-egr ; dnab-Gal4, UAS-CD8-GFP/puc-lacZ*
